# Diisopropyl {[(*R*)-2-(2-amino-6-chloro-9*H*-purin-9-yl)-1-methyl­eth­oxy]meth­yl}­phospho­nate

**DOI:** 10.1107/S1600536812006757

**Published:** 2012-03-03

**Authors:** Guobao Zhao, Xinhua He, Bohua Zhong

**Affiliations:** a Beijing Institute of Pharmacology and Toxicology, Beijing 100850, People’s Republic of China

## Abstract

In the title compound, C_15_H_25_ClN_5_O_4_P, the r.m.s. deviation for the purine ring system is 0.0165 Å. The coordination about the P atom is a distorted tetrahedron [O=P—O angles = 116.70 (6) and 109.87 (6)°]. In the crystal, molecules are linked by N—H⋯O hydrogen bonds, generating a three-dimensional network.

## Related literature

For details of the synthesis, see: Yu *et al.* (1992 ▶). For the bioactivity of nucleoside analogues, see: Martin (1989 ▶). For reference bond lengths, see: Allen *et al.* (1987 ▶). For a related structure, see: Baszczyňski *et al.* (2011 ▶).
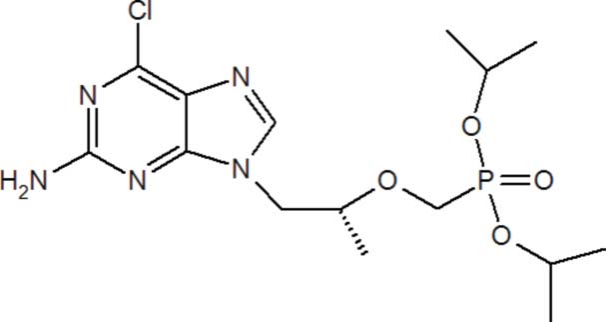


## Experimental

### 

#### Crystal data

C_15_H_25_ClN_5_O_4_P
*M* *_r_* = 405.82
Orthorhombic, 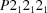

*a* = 7.7991 (12) Å
*b* = 13.950 (2) Å
*c* = 18.053 (3) Å
*V* = 1964.0 (6) Å^3^
*Z* = 4
Mo *K*α radiation
μ = 0.31 mm^−1^
*T* = 113 K
0.22 × 0.20 × 0.18 mm

#### Data collection

Rigaku Saturn CCD area-detector diffractometer
Absorption correction: multi-scan (*CrystalClear*; Rigaku, 2005 ▶) *T*_min_ = 0.936, *T*_max_ = 0.947
24716 measured reflections
4669 independent reflections
4468 reflections with *I* > 2σ(*I*)
*R*_int_ = 0.039

#### Refinement

*R*[*F* ^2^ > 2σ(*F* ^2^)] = 0.028
*wR*(*F* ^2^) = 0.066
*S* = 1.05
4669 reflections
240 parameters
H-atom parameters constrained
Δρ_max_ = 0.21 e Å^−3^
Δρ_min_ = −0.31 e Å^−3^
Absolute structure: Flack (1983 ▶), 2007 Friedel pairs
Flack parameter: 0.02 (4)

### 

Data collection: *CrystalClear* (Rigaku, 2005 ▶); cell refinement: *CrystalClear*; data reduction: *CrystalClear*; program(s) used to solve structure: *SHELXS97* (Sheldrick, 2008 ▶); program(s) used to refine structure: *SHELXL97* (Sheldrick, 2008 ▶); molecular graphics: *SHELXTL* (Sheldrick, 2008 ▶); software used to prepare material for publication: *CrystalStructure* (Rigaku, 2005 ▶).

## Supplementary Material

Crystal structure: contains datablock(s) I, global. DOI: 10.1107/S1600536812006757/fk2051sup1.cif

Structure factors: contains datablock(s) I. DOI: 10.1107/S1600536812006757/fk2051Isup2.hkl

Supplementary material file. DOI: 10.1107/S1600536812006757/fk2051Isup3.cml

Additional supplementary materials:  crystallographic information; 3D view; checkCIF report

## Figures and Tables

**Table 1:** Hydrogen-bond geometry (Å, °)

*D*—H⋯*A*	*D*—H	H⋯*A*	*D*⋯*A*	*D*—H⋯*A*
N5—H5*A*⋯O2^i^	0.89	2.13	3.0185 (16)	174
N5—H5*B*⋯O2^ii^	0.89	2.21	3.0920 (16)	172

Symmetry codes: (i) 
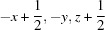
; (ii) 
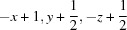
.

**Fig. 1.**
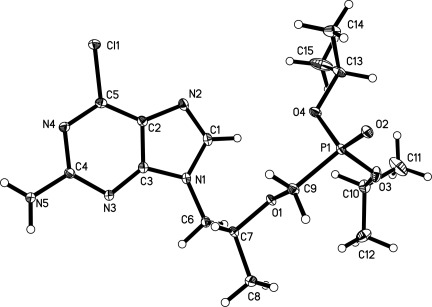
Molecular structure with displacement ellipsoids drawn at the 30% probability level and H atoms shown as small spheres of arbitrary radii.

**Fig. 2.**
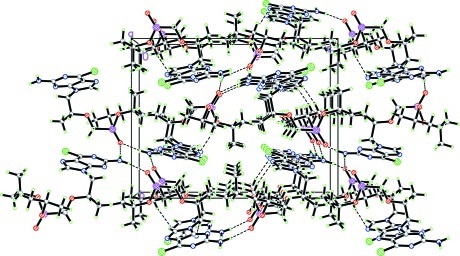
The crystal packing of (I) with hydrogen bonds drawn as dashed lines.

**Fig. 3.**
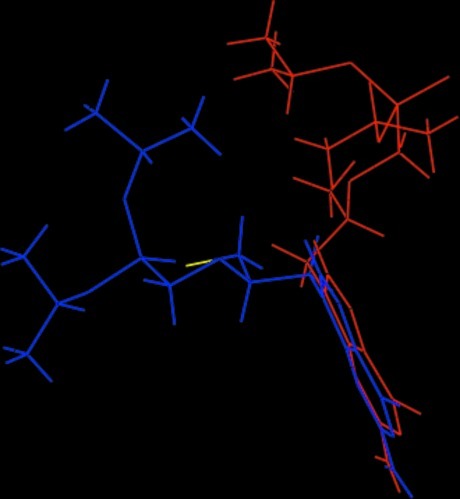
Superposition of the molecular conformations of the title compound (red) and its F-substituent (blue, Baszczyňski et al., 2011).

## supplementary crystallographic information

### Comment

Nucleoside analogues are in the lead in research and application of the anti-HBV drugs. In our recent work, the title compound (I), Fig. 1, was synthetized according to Kuo-Long Yu *et al.* (1992). As known, the molecular conformation in single-crystal is low energy conformation, and is helpful to quantitative structure-activity relationship (QSAR) study, therefore this structure determination was undertaken. Though the molecular structure of OSOWUP (Baszczyňski *et al.*, 2011) is similar to the title molecule, the molecular conformation is different (comparison is shown in Fig 3).

In (I), all bonds lengths and angles are normal (Allen *et al.*, 1987). Molecules are stacked along the *a* axis, and linked into a zigzag sheet propagating along the *c* axis by intermolecular N—H···O hydrogen bonds (Figure 2 and Table 2).

### Experimental

The title compound was synthesized according to the procedure of Kuo-Long Yu *et al.* (1992). Colourless single crystals (m.p. 404–406 K) were obtained by slow evaporation of a solution in absolute ethanol.

### Refinement

The H atoms linked to the C atoms were fixed geometrically and treated as riding with C—H = 0.95 Å (aromatic), 0.98Å (ethyl), 0.99 Å (methylene) with *U*_iso_(H) =1.2–1.5Ueq(C). H atoms of the amino group were located in a difference Fourier map and also refined riding with N-H = 0.89 Å.

### Crystal data

**Table:**

C_15_H_25_ClN_5_O_4_P	*D*_x_ = 1.372 Mg m^−^^3^
*M**_r_* = 405.82	Melting point: 404 K
Orthorhombic, *P*2_1_2_1_2_1_	Mo *K*α radiation, λ = 0.71070 Å
Hall symbol: P 2ac 2ab	Cell parameters from 7255 reflections
*a* = 7.7991 (12) Å	θ = 1.8–27.9°
*b* = 13.950 (2) Å	µ = 0.31 mm^−^^1^
*c* = 18.053 (3) Å	*T* = 113 K
*V* = 1964.0 (6) Å^3^	Block, colourless
*Z* = 4	0.22 × 0.20 × 0.18 mm
*F*(000) = 856	

### Data collection

**Table:**

Rigaku Saturn CCD area-detector diffractometer	4669 independent reflections
Radiation source: rotating anode	4468 reflections with *I* > 2σ(*I*)
Confocal monochromator	*R*_int_ = 0.039
Detector resolution: 7.31 pixels mm^-1^	θ_max_ = 27.9°, θ_min_ = 1.8°
ω and φ scans	*h* = −10→10
Absorption correction: multi-scan (*CrystalClear*; Rigaku, 2005)	*k* = −18→18
*T*_min_ = 0.936, *T*_max_ = 0.947	*l* = −21→23
24716 measured reflections	

### Refinement

**Table:**

Refinement on *F*^2^	Secondary atom site location: difference Fourier map
Least-squares matrix: full	Hydrogen site location: inferred from neighbouring sites
*R*[*F*^2^ > 2σ(*F*^2^)] = 0.028	H-atom parameters constrained
*wR*(*F*^2^) = 0.066	*w* = 1/[σ^2^(*F*_o_^2^) + (0.0412*P*)^2^ + 0.0557*P*] where *P* = (*F*_o_^2^ + 2*F*_c_^2^)/3
*S* = 1.05	(Δ/σ)_max_ = 0.001
4669 reflections	Δρ_max_ = 0.21 e Å^−^^3^
240 parameters	Δρ_min_ = −0.31 e Å^−^^3^
0 restraints	Absolute structure: Flack (1983), 2007 Friedel pairs
Primary atom site location: structure-invariant direct methods	Flack parameter: 0.02 (4)

### Special details

**Table:**

Geometry. All e.s.d.'s (except the e.s.d. in the dihedral angle between two l.s. planes) are estimated using the full covariance matrix. The cell e.s.d.'s are taken into account individually in the estimation of e.s.d.'s in distances, angles and torsion angles; correlations between e.s.d.'s in cell parameters are only used when they are defined by crystal symmetry. An approximate (isotropic) treatment of cell e.s.d.'s is used for estimating e.s.d.'s involving l.s. planes.
Refinement. Refinement of *F*^2^ against ALL reflections. The weighted *R*-factor *wR* and goodness of fit *S* are based on *F*^2^, conventional *R*-factors *R* are based on *F*, with *F* set to zero for negative *F*^2^. The threshold expression of *F*^2^ > σ(*F*^2^) is used only for calculating *R*-factors(gt) *etc*. and is not relevant to the choice of reflections for refinement. *R*-factors based on *F*^2^ are statistically about twice as large as those based on *F*, and *R*- factors based on ALL data will be even larger.

### Fractional atomic coordinates and isotropic or equivalent isotropic displacement parameters (Å^2^)

**Table:**

	*x*	*y*	*z*	*U*_iso_*/*U*_eq_	
Cl1	0.70932 (5)	0.28859 (3)	0.167353 (19)	0.02209 (9)	
P1	−0.06920 (4)	−0.07232 (3)	0.118252 (19)	0.01452 (8)	
O1	−0.02385 (12)	0.01978 (7)	0.24534 (5)	0.0159 (2)	
O2	−0.01664 (13)	−0.15893 (7)	0.07748 (5)	0.0181 (2)	
O3	−0.27016 (12)	−0.06894 (7)	0.12487 (6)	0.0235 (2)	
O4	−0.00844 (13)	0.02658 (7)	0.08574 (5)	0.0196 (2)	
N1	0.18098 (14)	0.18007 (8)	0.28806 (6)	0.0161 (2)	
N2	0.30287 (16)	0.23315 (8)	0.18180 (6)	0.0194 (3)	
N3	0.42068 (14)	0.19898 (8)	0.37275 (6)	0.0160 (2)	
N4	0.67530 (15)	0.25616 (9)	0.30921 (6)	0.0175 (2)	
N5	0.67325 (16)	0.23154 (9)	0.43524 (6)	0.0239 (3)	
H5A	0.6250	0.2149	0.4780	0.029*	
H5B	0.7770	0.2581	0.4341	0.029*	
C1	0.16466 (18)	0.19896 (10)	0.21332 (8)	0.0192 (3)	
H1	0.0612	0.1880	0.1868	0.023*	
C2	0.41989 (18)	0.23658 (9)	0.23985 (7)	0.0162 (3)	
C3	0.34601 (17)	0.20501 (9)	0.30642 (7)	0.0152 (3)	
C4	0.58455 (17)	0.22921 (10)	0.37114 (7)	0.0168 (3)	
C5	0.59151 (18)	0.25824 (9)	0.24618 (7)	0.0167 (3)	
C6	0.05519 (17)	0.13378 (10)	0.33578 (8)	0.0175 (3)	
H6A	−0.0582	0.1641	0.3282	0.021*	
H6B	0.0882	0.1433	0.3882	0.021*	
C7	0.04188 (18)	0.02758 (10)	0.31995 (7)	0.0176 (3)	
H7	0.1582	−0.0023	0.3227	0.021*	
C8	−0.0777 (2)	−0.02117 (12)	0.37504 (8)	0.0301 (4)	
H8B	−0.1886	0.0119	0.3753	0.045*	
H8C	−0.0270	−0.0185	0.4247	0.045*	
H8A	−0.0944	−0.0882	0.3606	0.045*	
C9	0.02123 (19)	−0.06768 (11)	0.20997 (7)	0.0187 (3)	
H9B	−0.0218	−0.1224	0.2396	0.022*	
H9A	0.1476	−0.0730	0.2069	0.022*	
C10	−0.36850 (19)	0.01452 (11)	0.15103 (8)	0.0212 (3)	
H10	−0.2899	0.0705	0.1578	0.025*	
C11	−0.4980 (3)	0.03695 (17)	0.09230 (11)	0.0483 (6)	
H11A	−0.4388	0.0548	0.0464	0.072*	
H11C	−0.5704	0.0903	0.1087	0.072*	
H11B	−0.5696	−0.0196	0.0833	0.072*	
C12	−0.4493 (2)	−0.01170 (11)	0.22435 (9)	0.0287 (4)	
H12B	−0.5299	−0.0648	0.2171	0.043*	
H12C	−0.5107	0.0439	0.2443	0.043*	
H12A	−0.3596	−0.0312	0.2593	0.043*	
C13	−0.0413 (2)	0.05138 (11)	0.00761 (8)	0.0237 (3)	
H13	−0.1452	0.0161	−0.0101	0.028*	
C14	0.1104 (2)	0.02249 (12)	−0.03813 (9)	0.0306 (4)	
H14C	0.2119	0.0579	−0.0215	0.046*	
H14B	0.0881	0.0372	−0.0903	0.046*	
H14A	0.1305	−0.0465	−0.0325	0.046*	
C15	−0.0760 (3)	0.15761 (14)	0.00579 (10)	0.0476 (6)	
H15C	−0.1792	0.1717	0.0348	0.071*	
H15A	−0.0933	0.1782	−0.0456	0.071*	
H15B	0.0220	0.1920	0.0270	0.071*	

### Atomic displacement parameters (Å^2^)

**Table:**

	*U*^11^	*U*^22^	*U*^33^	*U*^12^	*U*^13^	*U*^23^
Cl1	0.02487 (18)	0.02488 (17)	0.01653 (16)	−0.00578 (14)	0.00178 (13)	0.00367 (14)
P1	0.01522 (16)	0.01520 (16)	0.01314 (16)	0.00033 (13)	−0.00102 (14)	−0.00204 (13)
O1	0.0198 (5)	0.0169 (5)	0.0112 (4)	0.0001 (4)	−0.0030 (4)	−0.0028 (4)
O2	0.0199 (5)	0.0185 (5)	0.0160 (5)	0.0024 (4)	−0.0008 (4)	−0.0038 (4)
O3	0.0156 (5)	0.0207 (5)	0.0341 (6)	0.0004 (4)	−0.0005 (4)	−0.0124 (5)
O4	0.0278 (5)	0.0184 (5)	0.0126 (5)	−0.0008 (4)	−0.0008 (4)	0.0019 (4)
N1	0.0153 (6)	0.0163 (5)	0.0165 (6)	−0.0005 (5)	−0.0027 (5)	−0.0019 (4)
N2	0.0219 (6)	0.0180 (6)	0.0183 (6)	−0.0012 (5)	−0.0055 (5)	0.0011 (4)
N3	0.0155 (5)	0.0169 (6)	0.0155 (6)	−0.0012 (5)	−0.0009 (5)	−0.0006 (4)
N4	0.0168 (6)	0.0190 (6)	0.0168 (5)	−0.0013 (5)	0.0003 (5)	0.0033 (5)
N5	0.0177 (6)	0.0374 (7)	0.0166 (6)	−0.0083 (5)	−0.0022 (5)	0.0061 (5)
C1	0.0197 (7)	0.0174 (7)	0.0204 (7)	−0.0005 (6)	−0.0063 (6)	−0.0020 (5)
C2	0.0197 (7)	0.0139 (6)	0.0151 (6)	0.0001 (5)	−0.0020 (6)	0.0003 (5)
C3	0.0166 (6)	0.0110 (6)	0.0182 (6)	0.0009 (5)	−0.0011 (5)	−0.0012 (5)
C4	0.0171 (7)	0.0185 (7)	0.0148 (6)	0.0006 (5)	−0.0002 (6)	0.0004 (5)
C5	0.0218 (7)	0.0120 (6)	0.0162 (6)	0.0002 (5)	0.0013 (6)	0.0021 (5)
C6	0.0141 (6)	0.0228 (7)	0.0157 (6)	−0.0016 (5)	0.0003 (6)	−0.0043 (5)
C7	0.0186 (7)	0.0226 (7)	0.0116 (6)	−0.0020 (6)	−0.0030 (5)	−0.0016 (5)
C8	0.0366 (9)	0.0367 (9)	0.0168 (7)	−0.0163 (8)	0.0018 (7)	−0.0008 (7)
C9	0.0255 (7)	0.0153 (7)	0.0154 (7)	0.0006 (6)	−0.0012 (5)	0.0000 (6)
C10	0.0180 (7)	0.0207 (7)	0.0250 (8)	0.0058 (6)	0.0001 (6)	−0.0067 (6)
C11	0.0364 (10)	0.0772 (16)	0.0314 (10)	0.0307 (10)	−0.0098 (8)	−0.0162 (10)
C12	0.0283 (8)	0.0192 (8)	0.0385 (9)	0.0006 (6)	0.0108 (7)	0.0004 (7)
C13	0.0300 (8)	0.0298 (8)	0.0114 (7)	0.0036 (6)	−0.0026 (6)	0.0024 (6)
C14	0.0405 (10)	0.0264 (8)	0.0250 (8)	0.0026 (7)	0.0116 (7)	0.0028 (7)
C15	0.0765 (15)	0.0412 (11)	0.0250 (9)	0.0284 (11)	0.0135 (10)	0.0143 (8)

### Geometric parameters (Å, º)

**Table:**

Cl1—C5	1.7460 (14)	C6—H6B	0.9900
P1—O2	1.4729 (10)	C7—C8	1.523 (2)
P1—O4	1.5724 (11)	C7—H7	1.0000
P1—O3	1.5726 (10)	C8—H8B	0.9800
P1—C9	1.8009 (14)	C8—H8C	0.9800
O1—C9	1.4213 (17)	C8—H8A	0.9800
O1—C7	1.4453 (16)	C9—H9B	0.9900
O3—C10	1.4720 (16)	C9—H9A	0.9900
O4—C13	1.4748 (17)	C10—C11	1.498 (2)
N1—C3	1.3738 (17)	C10—C12	1.511 (2)
N1—C1	1.3806 (18)	C10—H10	1.0000
N1—C6	1.4566 (18)	C11—H11A	0.9800
N2—C1	1.3088 (19)	C11—H11C	0.9800
N2—C2	1.3904 (17)	C11—H11B	0.9800
N3—C3	1.3341 (17)	C12—H12B	0.9800
N3—C4	1.3462 (17)	C12—H12C	0.9800
N4—C5	1.3124 (17)	C12—H12A	0.9800
N4—C4	1.3756 (17)	C13—C14	1.498 (2)
N5—C4	1.3486 (17)	C13—C15	1.507 (2)
N5—H5A	0.8900	C13—H13	1.0000
N5—H5B	0.8901	C14—H14C	0.9800
C1—H1	0.9500	C14—H14B	0.9800
C2—C5	1.377 (2)	C14—H14A	0.9800
C2—C3	1.4038 (19)	C15—H15C	0.9800
C6—C7	1.5123 (19)	C15—H15A	0.9800
C6—H6A	0.9900	C15—H15B	0.9800
			
O2—P1—O4	116.70 (6)	H8B—C8—H8C	109.5
O2—P1—O3	109.87 (6)	C7—C8—H8A	109.5
O4—P1—O3	107.60 (6)	H8B—C8—H8A	109.5
O2—P1—C9	112.33 (6)	H8C—C8—H8A	109.5
O4—P1—C9	101.17 (6)	O1—C9—P1	110.30 (9)
O3—P1—C9	108.63 (6)	O1—C9—H9B	109.6
C9—O1—C7	113.30 (10)	P1—C9—H9B	109.6
C10—O3—P1	124.57 (9)	O1—C9—H9A	109.6
C13—O4—P1	120.69 (9)	P1—C9—H9A	109.6
C3—N1—C1	105.90 (11)	H9B—C9—H9A	108.1
C3—N1—C6	126.91 (12)	O3—C10—C11	106.83 (12)
C1—N1—C6	126.91 (11)	O3—C10—C12	107.87 (12)
C1—N2—C2	103.04 (11)	C11—C10—C12	112.93 (15)
C3—N3—C4	112.04 (11)	O3—C10—H10	109.7
C5—N4—C4	117.03 (12)	C11—C10—H10	109.7
C4—N5—H5A	121.4	C12—C10—H10	109.7
C4—N5—H5B	117.2	C10—C11—H11A	109.5
H5A—N5—H5B	120.9	C10—C11—H11C	109.5
N2—C1—N1	114.73 (12)	H11A—C11—H11C	109.5
N2—C1—H1	122.6	C10—C11—H11B	109.5
N1—C1—H1	122.6	H11A—C11—H11B	109.5
C5—C2—N2	135.10 (12)	H11C—C11—H11B	109.5
C5—C2—C3	113.37 (12)	C10—C12—H12B	109.5
N2—C2—C3	111.41 (12)	C10—C12—H12C	109.5
N3—C3—N1	127.56 (13)	H12B—C12—H12C	109.5
N3—C3—C2	127.52 (13)	C10—C12—H12A	109.5
N1—C3—C2	104.91 (12)	H12B—C12—H12A	109.5
N3—C4—N5	118.43 (12)	H12C—C12—H12A	109.5
N3—C4—N4	126.27 (12)	O4—C13—C14	109.06 (12)
N5—C4—N4	115.27 (12)	O4—C13—C15	106.42 (13)
N4—C5—C2	123.45 (12)	C14—C13—C15	113.22 (14)
N4—C5—Cl1	116.74 (10)	O4—C13—H13	109.4
C2—C5—Cl1	119.80 (10)	C14—C13—H13	109.4
N1—C6—C7	111.64 (11)	C15—C13—H13	109.4
N1—C6—H6A	109.3	C13—C14—H14C	109.5
C7—C6—H6A	109.3	C13—C14—H14B	109.5
N1—C6—H6B	109.3	H14C—C14—H14B	109.5
C7—C6—H6B	109.3	C13—C14—H14A	109.5
H6A—C6—H6B	108.0	H14C—C14—H14A	109.5
O1—C7—C6	105.91 (11)	H14B—C14—H14A	109.5
O1—C7—C8	110.96 (11)	C13—C15—H15C	109.5
C6—C7—C8	110.85 (12)	C13—C15—H15A	109.5
O1—C7—H7	109.7	H15C—C15—H15A	109.5
C6—C7—H7	109.7	C13—C15—H15B	109.5
C8—C7—H7	109.7	H15C—C15—H15B	109.5
C7—C8—H8B	109.5	H15A—C15—H15B	109.5
C7—C8—H8C	109.5		
			
O2—P1—O3—C10	−169.42 (10)	C5—N4—C4—N3	4.4 (2)
O4—P1—O3—C10	−41.40 (12)	C5—N4—C4—N5	−177.53 (13)
C9—P1—O3—C10	67.35 (12)	C4—N4—C5—C2	1.1 (2)
O2—P1—O4—C13	52.62 (12)	C4—N4—C5—Cl1	−177.90 (10)
O3—P1—O4—C13	−71.35 (11)	N2—C2—C5—N4	−179.99 (14)
C9—P1—O4—C13	174.81 (10)	C3—C2—C5—N4	−4.5 (2)
C2—N2—C1—N1	0.42 (16)	N2—C2—C5—Cl1	−1.0 (2)
C3—N1—C1—N2	0.27 (16)	C3—C2—C5—Cl1	174.40 (10)
C6—N1—C1—N2	−173.85 (13)	C3—N1—C6—C7	−100.58 (15)
C1—N2—C2—C5	174.56 (15)	C1—N1—C6—C7	72.34 (17)
C1—N2—C2—C3	−0.96 (15)	C9—O1—C7—C6	154.99 (11)
C4—N3—C3—N1	179.73 (13)	C9—O1—C7—C8	−84.65 (15)
C4—N3—C3—C2	1.00 (19)	N1—C6—C7—O1	−64.84 (14)
C1—N1—C3—N3	−179.77 (13)	N1—C6—C7—C8	174.73 (12)
C6—N1—C3—N3	−5.7 (2)	C7—O1—C9—P1	178.87 (9)
C1—N1—C3—C2	−0.82 (14)	O2—P1—C9—O1	176.25 (9)
C6—N1—C3—C2	173.30 (12)	O4—P1—C9—O1	51.07 (10)
C5—C2—C3—N3	3.5 (2)	O3—P1—C9—O1	−62.00 (11)
N2—C2—C3—N3	−179.91 (13)	P1—O3—C10—C11	125.75 (14)
C5—C2—C3—N1	−175.42 (11)	P1—O3—C10—C12	−112.57 (13)
N2—C2—C3—N1	1.13 (15)	P1—O4—C13—C14	−92.90 (14)
C3—N3—C4—N5	176.77 (12)	P1—O4—C13—C15	144.64 (13)
C3—N3—C4—N4	−5.24 (19)		

### Hydrogen-bond geometry (Å, º)

**Table:**

*D*—H···*A*	*D*—H	H···*A*	*D*···*A*	*D*—H···*A*
N5—H5*A*···O2^i^	0.89	2.13	3.0185 (16)	174
N5—H5*B*···O2^ii^	0.89	2.21	3.0920 (16)	172

Symmetry codes: (i) −*x*+1/2, −*y*, *z*+1/2; (ii) −*x*+1, *y*+1/2, −*z*+1/2.
